# Multifunctional PdH-hydride MOFs for synergistic hydrogen and photothermal antibacterial therapy in accelerated wound healing

**DOI:** 10.3389/fphar.2025.1587890

**Published:** 2025-04-30

**Authors:** Qing Wang, Daixing Zhang, Yining Qi, Changbao Huang, Dejun Ding, Chuanliang Liu

**Affiliations:** ^1^ School of Clinical Medicine, Shandong Second Medical University, Weifang, Shandong, China; ^2^ College of Pharmacy, Shandong Second Medical University, Weifang, Shandong, China; ^3^ Department of Geriatrics, Weifang People’s Hospital, Weifang, Shandong, China

**Keywords:** antibacterial therapy, hydrogen therapy, photothermal therapy, wound healing, hydride MOFs

## Abstract

**Introduction:**

The growing threat of bacterial infections poses a critical challenge to public health, underscoring the urgent need for innovative antibacterial agents and therapeutic strategies. In response, we have developed a multifunctional nanoplatform based on palladium-hydride metal–organic frameworks (P(H)ZPAg) for synergistic hydrogen and photothermal antibacterial therapy.

**Methods:**

This nanoplatform integrates palladium hydride (PdH) encapsulated within a zeolitic imidazolate framework (ZIF-8), surface modification with polydopamine (PDA), and in situ generation of silver nanoparticles (Ag NPs) to achieve enhanced antibacterial efficacy. Comprehensive characterization was performed to assess hydrogen release kinetics, photothermal performance, and silver-mediated bactericidal activity. The therapeutic potential of P(H)ZPAg was further evaluated in vivo using a *Staphylococcus aureus*-infected rat wound model.

**Results:**

The P(H)ZPAg nanoplatform demonstrated a successful combination of hydrogen release, photothermal conversion, and silver ion-based antibacterial mechanisms. In vitro assays revealed potent synergistic antibacterial effects against both *Escherichia coli* and *Staphylococcus aureus*. In vivo studies showed that treatment with P(H)ZPAg nanoparticles significantly enhanced wound healing and bacterial clearance compared to control groups.

**Discussion:**

These findings highlight the potential of combining hydrogen therapy, photothermal therapy, and silver ion release within a single nanoplatform to markedly improve antibacterial outcomes. This study presents a promising strategy for the development of multifunctional nanotherapeutics, offering a novel and effective approach for managing topical bacterial infections and promoting wound healing.

## 1 Introduction

Skin damage, encompassing both acute and chronic wounds, represents a widespread and significant concern in clinical practice (Falanga et al., 2022; Siddiqui and Bernstein, 2010). Of particular concern are trauma-induced infections caused by pathogenic bacteria, which continue to pose substantial challenges to global public health (Falcone et al., 2021). It is estimated that approximately 15 million people die annually worldwide as a result of bacterial infections (Fongang et al., 2023). These infections can severely impede the wound healing process by inducing chronic inflammation, a hallmark of non-healing wounds. For decades, antibiotics have served as the cornerstone in the treatment of bacterial infections. However, the overuse and misuse of these drugs have inadvertently contributed to the rise of antibiotic-resistant pathogens. This growing resistance undermines the efficacy of current therapeutic options, thereby exacerbating the challenge of managing bacterial infections (MacNair et al., 2024). As such, it is crucial to develop new strategies for combating bacterial infections and promoting wound healing while minimizing reliance on antibiotics.

In recent decades, considerable efforts have been directed toward the development of alternative therapeutic strategies to address antibiotic resistance, including the use of thermal effects (Chen et al., 2020; Huo et al., 2021), reactive oxygen species (ROS) (Lam et al., 2020; Li H. et al., 2021), metal ions (Frei et al., 2023), cationic ions (Cheng et al., 2022) and quaternary ammonium ions (Mohapatra et al., 2023). Among these innovative approaches, gas therapy has emerged as a novel and promising treatment paradigm, garnering significant attention for its potential advantages over traditional therapies (Wang T.-Y. et al., 2023; Wang Z. et al., 2023). Notably, gas therapy offers the ability to circumvent the development of drug resistance, which remains a persistent challenge in infection management. Moreover, gaseous molecules serve as vital endogenous signaling agents, playing crucial roles in various biological processes. Consequently, gas therapy is considered an environmentally friendly (“green”) treatment option, providing targeted therapeutic effects with minimal toxicity to healthy tissues. To date, several gaseous molecules, such as nitric oxide (NO) (Andrabi et al., 2023), carbon monoxide (CO) (Cheng and Hu, 2021), hydrogen sulfide (H_2_S) (Shi et al., 2024), and hydrogen (H_2_) (Yang et al., 2020; Ye et al., 2024), have been extensively studied and shown to be effective against a wide range of bacterial strains, including multidrug-resistant (MDR) bacteria. Notably, hydrogen, the smallest gas with strong reducing properties, is regarded as a highly biocompatible and environmentally friendly gas (Yang et al., 2020). It demonstrates exceptional safety, even at high concentrations, without causing damage to normal cells or tissues. Furthermore, active H_2_ has been shown to significantly enhance antibacterial and wound-healing applications by regulating the expression of genes involved in bacterial metabolism (Benoit Stéphane et al., 2020). For instance, Xue et al. (Yu et al., 2019) synthesized Pd nanocubes and incorporated H_2_ into their lattice, creating PdH nano-hydrides for antibacterial and wound-healing therapies. More importantly, H_2_ molecules can disrupt bacterial membrane permeability, thereby facilitating the entry of antibacterial agents into bacterial cells, promoting the leakage of cellular contents, and ultimately leading to bacterial death (Li et al., 2023). However, hydrogen therapy applied alone is often limited by its low tissue permeability, poor solubility, and suboptimal bioavailability (Jiang et al., 2024). Therefore, combining controlled hydrogen release with other therapeutic modalities can create a synergistic antibacterial effect, significantly enhancing the overall antibacterial performance. Near-infrared (NIR) light-assisted bactericidal strategies, such as photothermal therapy (PTT), have emerged as promising alternatives due to their operability, safety, and controllability (Chen et al., 2020; Huo et al., 2021). Photoconversion agents, including metal nanoparticles (Kadkhoda et al., 2022), metal-organic frameworks (MOFs) (Zheng et al., 2021), and carbon dots (Li B. et al., 2021), can effectively convert light into thermal energy, which can be used to eradicate bacteria. This hyperthermic effect disrupts bacterial cell membranes, denatures proteins, enzymes, and DNA, and induces cell death (Chen et al., 2020). Polydopamine (PDA) nanoparticles, in particular, have recently been utilized in antibacterial studies due to their excellent biocompatibility and efficient photothermal conversion properties (Yang et al., 2021). Qi et al. (2025) proposed an inorganic-organic hybrid system composed of a zeolitic imidazolate framework (ZIF-8) and PDA, which leverages 808 nm near-infrared-induced photothermal effects to enhance catalytic activity for the treatment of bacteria-infected pressure ulcers. Additionally, metal or metal oxide nanocomposites (Pachaiappan et al., 2021), have proven to be powerful antimicrobial agents, exhibiting broad-spectrum activity against both Gram-positive and Gram-negative microorganisms. These composites frequently release metal ions that bind to and interact with bacterial proteins and enzymes, generating ROS that cause structural damage to bacterial cell walls and membranes, thereby leading to membrane permeability alterations, cellular disintegration, and bacterial death (Godoy-Gallardo et al., 2021). Therefore, integrating hydrogen release with silver nanoparticles (AgNPs) and PTT capabilities is expected to synergistically enhance the antibacterial efficacy of these therapies.

**SCHEME 1:**
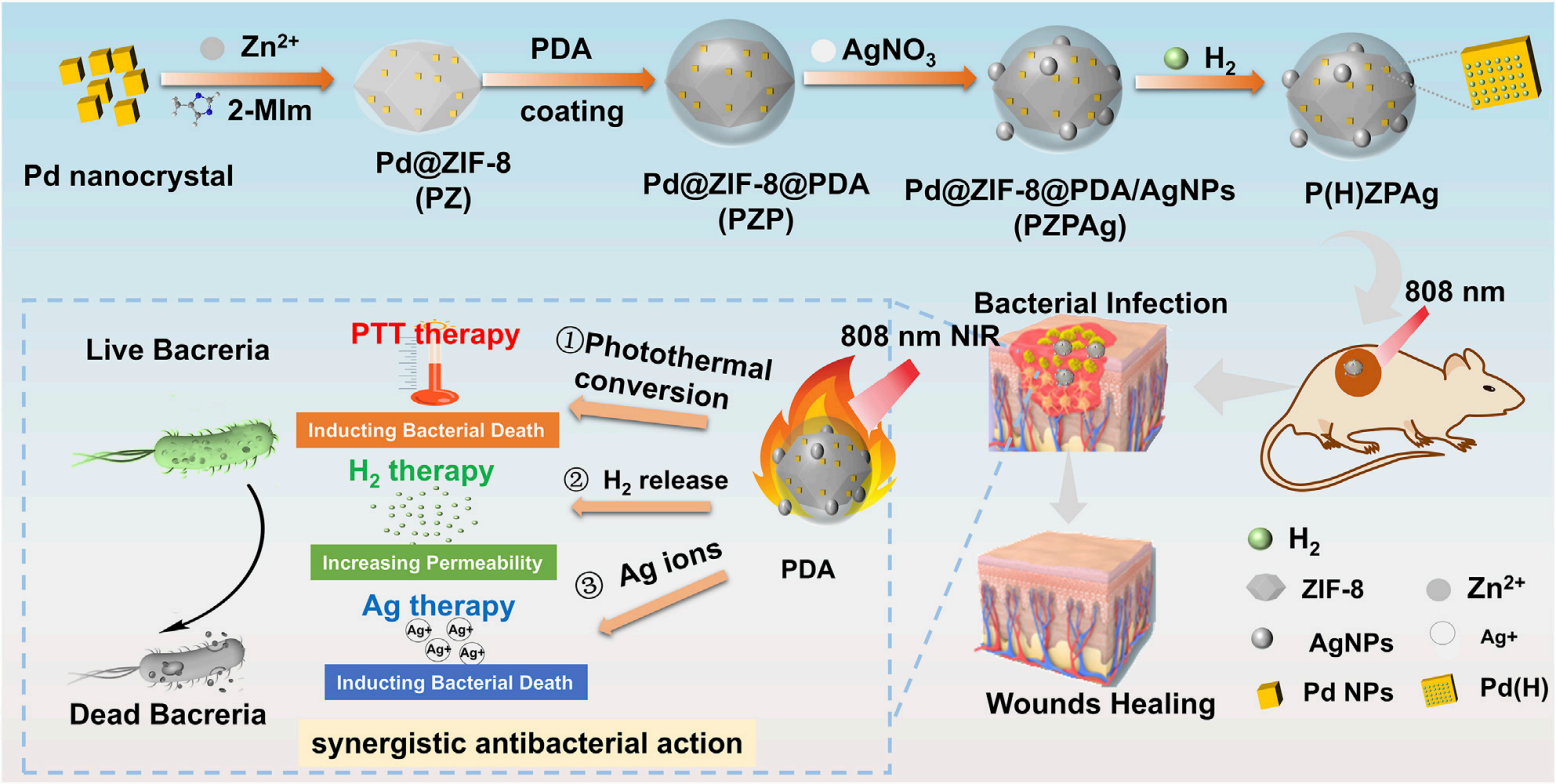
Illustration of the P(H)ZPAg NPs synthesis and its multimode synergistic mechanism for antibacterial and promoting healing process for bacterial infected wounds.

In this study, a multifunctional PdH-hydride MOFs-based nanoplatform (PdH@ZIF@PDA/Ag nanoparticles, denoted as P(H)ZPAg NPs) was developed to integrate hydrogen release, PTT, and AgNP therapy for the treatment of bacterial infections associated with wound healing (Scheme 1), achieving a synergistic antibacterial effect that surpasses conventional single-modality treatments. Mechanistically, the released hydrogen was found to compromise the permeability of bacterial cell membranes, thereby facilitating the enhanced entry of Ag^+^ ions (Zhang et al., 2022). In addition, PTT contributed to bacterial eradication by generating localized thermal energy that disrupts microbial structures and induces irreversible cellular damage, collectively leading to significantly improved antibacterial efficacy (Yang et al., 2021). ZIF-8 was initially used to encapsulate PdH through a simple covalent growth strategy, preserving the functionality of PdH as a hydrogen-releasing material. Subsequently, PDA was modified onto the surface of PdH@ZIF via mussel-inspired chemistry. In this system, PDA serves as an efficient NIR-absorbing agent with excellent biocompatibility and photothermal stability, facilitating PTT. Furthermore, AgNPs were deposited onto the surface of PDA through coordination forces and *in situ* reduction by the catechol groups of PDA. The resulting P(H)ZPAg NPs exhibit three distinct features: (i) high photothermal performance of PDA, leading to effective photothermal antibacterial therapy; (ii) hydrogen-releasing PdH-Hydride MOFs that demonstrate on-demand, controlled hydrogen release under NIR laser irradiation; and (iii) the release of Ag^+^ ions, which further promote bacterial death. *In vitro* antibacterial efficacy and *in vivo* wound disinfection were evaluated using *Escherichia coli* (*E. coli*) and *Staphylococcus aureus* (*S. aureus*) bacteria, as well as bacteria-bearing mouse models. The antibacterial system demonstrated enhanced antibacterial activity, offering a promising strategy in the fight against bacterial infections.

## 2 Materials and methods

### 2.1 Materials

Poly (vinyl pyrrolidone) (PVP), L-ascorbic acid, 2-methylimidazolate (2-MIm), dopamine hydrochloride (DA) were purchased from Shanghai Aladdin Biochemical Technology Co. KBr, sodium tetrachloropalladate (Na_2_PdCl_4_), zinc nitrate hexahydrate (Zn(NO_3_)_2_·6H_2_O), sodium borohydride (NaBH_4_), and silver nitrate (AgNO_3_) were purchased from Shanghai McLean Biochemistry and Technology Co. Methylene blue, 2,5-diphenyltetra-zolium bromide (MTT), 4′,6-diamidino-2-phenylindole (DAPI), were purchased from Shanghai Macklin Biochemical Co., Ltd. Phosphate buffered saline (PBS) and 2,2-diphenyl-1-picrylhydrazyl (DPPH) radical scavenging assay was purchased from Beijing Solaibao Technology Co. LB broth powder and agar powder were purchased from Qingdao Hi-Tech Industrial Park HaiBo Biotechnology Co. Polyformaldehyde tissue fixation solution (4%) was purchased from Biosharp Biological Technology (Anhui, China). Live & Dead Bacterial Staining Kit was provided by Yeasen Biotechnology (Shanghai) Co., Ltd.

### 2.2 Synthesis of Pd@Zif-8 (PZ) NPs

PZ NPs were obtained referring to reported methods (Zhang et al., 2022). In a typical synthesis of Pd nanocubes, 11 mL of an aqueous solution containing PVP, L-ascorbic acid (60 mg), potassium bromide (KBr, 300 mg), and sodium tetrachloropalladate (Na_2_PdCl_4_, 57 mg) was heated to 80°C in air under continuous magnetic stirring for 3 h. After the reaction, the solution was allowed to cool to room temperature. The resulting Pd nanocubes were isolated via centrifugation and subsequently washed three times with a water/acetone mixture. The purified Pd nanoparticles were then dispersed in 10 mL of deionized water and stored at 4°C in the dark to maintain stability. These Pd nanoparticles were subsequently encapsulated within a ZIF-8 using a previously reported method (Zhang et al., 2022). Briefly, 200 mg of zinc nitrate hexahydrate (Zn(NO_3_)_2_·6H_2_O) was completely dissolved in 2.8 mL of deionized water. Next, 2 mL of the synthesized Pd nanocubes solution was added to the zinc nitrate solution, and the mixture was magnetically stirred for 10 min. Next, 2 g of 2-MIm was dissolved in 8 mL of deionized water to form a homogeneous solution, which was then rapidly added to the above Pd nanocubes mixture. The combined solution was stirred continuously for an additional 12 min. The final product was washed three times with a 1:1 ethanol-water solution by centrifugation at 8,000 rpm and subsequently freeze-dried to yield PZ NPs.

### 2.3 Synthesis Pd@Zif-8@PDA (PZP) and Pd@Zif-8@PDA/Ag (PZPAg) NPs

Firstly, 100 mg of PZ NPs were uniformly redispersed in 20 mL of tris-buffer solution (pH 8.5) using sonication for 10 min to achieve a homogeneous suspension. Subsequently, 50 mg of DA was introduced into the solution, and the mixture was subjected to continuous stirring for 48 h to facilitate the reaction. The resulting product, denoted as PZP NPs, was thoroughly washed three times with ethanol to remove any unreacted species and then redispersed in 20 mL of methanol. Following this, 100 mL of an aqueous silver nitrate (AgNO_3_) solution (8 mg mL^−1^) was added dropwise to the suspension at room temperature, and the reaction was allowed to proceed for 1 h. The final product, PZPAg, characterized by its distinct brown coloration, was collected through washing and freeze-drying overnight to yield a stable powder.

### 2.4 Synthesis P(H)ZPAg NPs

A solution (5 mL) of PZPAg NPs at a concentration of 5 mg/mL, which was subsequently transferred into a 20 mL vial and securely sealed with a rubber stopper. In parallel, 100 mg of sodium borohydride (NaBH_4_) was measured and placed into a separate vial, which was also sealed with a rubber stopper. The two vials were then interconnected using a capillary tube. To facilitate atmospheric connection, a needle attached to a 1 mL syringe was inserted into the first vial containing the PZPAg NPs. Following this, 2 mL of a sulfuric acid solution with a pH of 5 was injected into the second vial containing NaBH_4_. The acidic environment induced the generation of hydrogen gas from NaBH_4_, which subsequently diffused through the capillary into the vial containing the Pd tetrahedrons solution. After a reaction period of 15 min, the needle and capillary were carefully removed. The vial containing the P(H)ZPAg NPs was then sealed and stored in a dark environment to prevent photodegradation, ensuring its stability for subsequent applications.

### 2.5 Characterization

The morphological characteristics of the samples were examined using a Hitachi HT-7700 transmission electron microscope (Hitachi, Japan). The hydrodynamic diameter and ζ-potential of the nanoparticles were quantitatively assessed using a Zetasizer Nano ZS90 instrument (Malvern Instruments, United Kingdom), providing insights into their colloidal stability and surface charge properties. Chemical composition and elemental analysis were conducted via X-ray photoelectron spectroscopy (XPS) employing an AXIS UltraDLD spectrometer (Kratos Analytical, Japan).

### 2.6 *In vitro* photothermal performance of PZPAg NPs

The photothermal characteristics of the PZPAg NPs were systematically investigated using an 808 nm fiber optic coupler. Under NIR light irradiation (808 nm, 0.75 W cm^−2^) for a duration of 6 min, the temperature changes of PZPAg NP dispersions at varying concentrations (0, 50, 100, 150, 200, and 250 μg mL^−1^) were meticulously monitored. Real-time temperature data and thermal imaging were captured using an infrared thermal camera, providing a comprehensive visualization of the photothermal response. In NIR light absorption density experiment, PZPAg NPs at a fixed concentration of 200 μg/mL were subjected to NIR light irradiation at different power densities (0.35, 0.5, and 0.75 W cm^−2^) for 6 min to evaluate the influence of power intensity on photothermal performance. Furthermore, the photothermal stability of the PZPAg NPs (200 μg/mL) was assessed over five consecutive irradiation cycles (808 nm, 0.75 W cm^−2^), demonstrating their robustness under repeated exposure. The photothermal conversion efficiency (η) was calculated in accordance with established methodologies (Cui et al., 2023):

$$\eta =\frac{hA\left(T_{\max,sam}-T_{\max,water}\right)}{I\left(1-10^{-A_{808}}\right)}$$ (1)

### 2.7 Reductive hydrogen release measurement in solution

Prior to conducting the experiment, a standard calibration curve for methylene blue (MB) was established using a UV–vis spectrophotometer. The characteristic absorption peak of MB at 664 nm was measured for aqueous solutions of varying concentrations, and the resulting data were used to construct the calibration curve (Supplementary Figure S1). This curve served as a reference for quantifying the concentration of reductive hydrogen released during the reaction. Additionally, hydrogen-rich water was prepared as a control by saturating deionized water with hydrogen gas generated in the aforementioned reaction apparatus. To evaluate the catalytic activity of the P(H)ZPAg NPs and assess the release of reductive hydrogen from the nanohydride systems, the reduction of MB was employed as a model reaction. For this purpose, 3.4 mL of MB solution (25 µM) was combined with 50 µL of P(H)ZPAg NPs (1 mg mL^−1^) and 50 µL of deionized water in a sealed 1 cm quartz cuvette. In a parallel experiment, 50 µL of PZPAg NPs (1 mg mL^−1^) was mixed with 50 µL of hydrogen-rich water and added to an identical MB solution. The reduction process was monitored in real time by tracking the characteristic absorption of MB at 664 nm using the UV–vis spectrophotometer. Measurements were recorded continuously until the absorbance reached a steady state, indicating the completion of the reaction.

### 2.8 Intracellular reductive hydrogen release

The intracellular release of hydrogen (H_2_) from P(H)ZPAg NPs was assessed using a methylene blue-platinum (MB-Pt) probe (Chen et al., 2021). For this analysis, L929 cells were seeded into 6-well plates and incubated for 24 h. Following this, the MB-Pt probe was introduced into the co-culture system and allowed to incubate for 4 h. The culture medium was then replaced with a fresh medium containing P(H)ZPAg NPs nanohydride at a concentration of 50 μg mL^−1^. Additionally, the mixture was subjected to NIR light irradiation (808 nm, 0.75 W cm^−2^) for a duration of 6 min, to evaluate the influence of NIR irradiation. The hydrogen release was monitored at specific time intervals (0, 30, and 60 min). Observations of color changes were conducted using an inverted fluorescence microscope (Olympus, Japan).

### 2.9 DPPH and hydroxyl radicals scavenging activity

The *in vitro* free radical scavenging efficacy of hydrogels was assessed using two distinct assays: the DPPH radical scavenging assay and the hydroxyl radical scavenging assay. The DPPH radical scavenging activity was evaluated following a modified protocol (Liu et al., 2019). Briefly, 3 mL of a 100 mM DPPH solution prepared in 95% ethanol was introduced into a scintillation vial. Subsequently, NPs samples with concentrations ranging from 0.2 to 1 mg mL^−1^ were added to the solution. The mixture was kept in the dark for 30 min, after which the decrease in absorbance at 517 nm was measured to determine the scavenging activity. The DPPH radical scavenging activity was quantified using the following equation (Liu et al., 2019):

$$Radical\;Scavenging\;Activity\;\left(\%\right)=\left[1-\frac{\left(\text{Ai}–\text{Aj}\right)}{Ac}\right]\times 100\%$$ (2)

where Ac is the absorbance of DPPH solution without particle samples, Ai is the absorbance of the sample particles mixed with DPPH solution, and Aj is the absorbance of the sample particles mixed with 95% ethanol.

The hydroxyl radical scavenging activity was evaluated using a commercially available hydroxyl radical assay kit (Nanjing Jiancheng Bioengineering Institute, Nanjing, China), which operates on the principle of the Fenton reaction (Fu et al., 2023). According to the procedure, NPs samples of varying concentrations were incubated at 37°C for 1 min. Subsequently, Fenton reagent was introduced into each well, and the mixture was allowed to stand for 20 min. The absorbance at 550 nm was then measured using a microplate reader. A control experiment was conducted by substituting H_2_O_2_ with distilled water. The hydroxyl radical scavenging activity was quantified using the following equation:

$$Radical\;Scavenging\;Activity\;\left(\%\right)=\left[\frac{\left(A_{0}–A_{1}\right)}{A_{0}}\right]\times 100\%$$ (3)

where A_0_ is the absorbance of the control, and A_1_ is the absorbance of the sample.

### 2.10 *In vitro* antibacterial assay

The antibacterial efficacy of the NPs was investigated against *S. aureus* and *E. coli* using the spread plate method. Luria Broth (LB) medium served as the culture medium for both bacterial strains. The experimental design comprised six groups: (1) Control, (2) P(H)Z NPs, (3) PZP NPs, (4) P(H)ZP NPs, (5) PZPAg, and (6) P(H)ZPAg. To assess the antibacterial efficacy of the nanoparticles (NPs) under photothermal conditions, a standardized bacterial suspension was prepared at a concentration of 10^6^ colony-forming units per milliliter (CFU mL^−1^). A 0.1 mL aliquot of this suspension was mixed with an equal volume (0.1 mL) of the respective NP solution at a concentration of 400 μg mL^−1^, ensuring uniform interaction between the bacterial cells and the NPs. The mixtures were subjected to NIR laser irradiation at a power density of 0.75 W cm^−2^ for 6 min. Following treatment, all bacterial suspensions were incubated at 37°C for 12 h under optimal growth conditions to allow for potential bacterial recovery or further inhibition. Subsequently, the samples were serially diluted in sterile phosphate-buffered saline (PBS) to achieve appropriate countable colony densities. After an additional 24-h incubation period at 37°C, bacterial colonies were clearly visible, and high-resolution images of the plates were captured for quantitative analysis. Colony enumeration was performed using ImageJ software (National Institutes of Health, United States), employing standardized thresholding and particle-counting algorithms to ensure accuracy. The bacterial inhibition rate (%) was calculated by comparing the colony-forming units (CFUs) of the treated groups against the untreated control, providing a quantitative measure of the NPs’ antibacterial efficiency with or without NIR.

To further elucidate the antibacterial mechanisms, a live/dead staining assay was performed. After treatment, 100 μL of the bacterial suspension was transferred to a 96-well plate, and 1 μL of a dual-fluorescence dye mixture (DMAO/EthD-III) was added to each well. The plate was incubated in the dark at room temperature for 15 min. Bacterial viability was assessed using an inverted fluorescence microscope (Leica DMI4000B). Viable cells were stained green by DMAO, while non-viable or membrane-compromised cells were stained red by EthD-III. Fluorescence images were captured to quantify the proportion of live and dead cells.

### 2.11 Animal model

Five-week-old BALB/c mice (weighing 14–18 g) were procured from Jinan Pengyue Experimental Animal Co., Ltd. The mice were acclimatized for 7 days in standard plastic rodent cages under controlled environmental conditions prior to experimentation. To establish a wound infection model, the mice were anesthetized using pentobarbital sodium, and a circular wound (5 mm in diameter) was created on the disinfected dorsal skin using a sterile annulus. The wound was inoculated with *S. aureus* (100 μL, 10^7^ CFU mL^−1^). The mice were subsequently divided into experimental groups and treated with various formulations, including PBS (control), NIR, PZP, P(H)ZP, P(H)ZPAg, and P(H)ZPAg combined with near-infrared irradiation (0.75 W cm^−2^ for 6 min), to assess their antibacterial efficacy in promoting wound healing. Wound progression was documented using a digital camera on days 0, 3, 7, and 12 post-treatments. After 12 days, the mice were euthanized, and skin tissue surrounding the wound was excised, fixed in 4% paraformaldehyde, and embedded in paraffin for histological analysis. Hematoxylin and eosin (H&E) staining and Masson’s trichrome staining were performed to evaluate tissue regeneration and collagen deposition, respectively.

To assess the *in vivo* photothermal performance of the P(H)ZPAg, an 808 nm fiber optic coupler (0.75 W cm^−2^) was employed in conjunction with a FLIR thermal imaging camera to monitor temperature changes over a 6-min irradiation period. Additionally, to quantify the antibacterial efficacy, wound tissue samples were collected from each treatment group on the third day. The bacterial solutions were serially diluted, plated onto LB agar plates, and incubated at 37°C for 24 h. Colony-forming units were enumerated using ImageJ software, and the bacterial inhibition rate was calculated.

### 2.12 Biocompatibility, blood compatibility, and biosafety evaluation

The *in vitro* cytotoxicity of P(H)ZPAg NPs was evaluated using the MTT assay. Initially, L929 cells were seeded into 96-well plates and allowed to adhere for 24 h. Following this, the culture medium was replaced with basal medium containing varying concentrations of P(H)ZPAg NPs (0–400 μg/mL). After incubation, a standard MTT assay was performed to determine cell viability. To further corroborate these findings, a live/dead cell staining assay was conducted under identical treatment conditions. Fluorescence images were acquired using an inverted fluorescence microscope (Leica DMI4000B).

For the hemolysis assay, fresh mouse blood was utilized. Red blood cells (RBCs) were isolated via centrifugation (1,000 rpm, 5 min) and washed six times with phosphate-buffered saline (PBS; 0.01 M, pH 7.4). A 0.5 mL aliquot of RBC suspension in PBS was mixed with 0.5 mL of PZ, PZP, or PZPAg NPs (400 μg/mL). PBS and deionized water served as negative and positive controls, respectively. The mixtures were incubated at 37°C for 4 h, followed by centrifugation to observe the supernatant’s color. Hemolysis was quantified by measuring the absorbance of the supernatant using an ultraviolet-visible spectrophotometer.

Biosafety was further assessed through *in vivo* antimicrobial experiments in mice. Body weight changes were monitored throughout the treatment period to evaluate systemic toxicity. Upon completion of the wound healing experiments, the mice were euthanized via cervical dislocation. Major organs, including the heart, liver, spleen, lungs, and kidneys, were excised and fixed in 4% paraformaldehyde. The tissues were paraffin-embedded, sectioned at 5 μm thickness, and subjected to H&E staining for histopathological analysis.

### 2.13 Statistical analysis

All experiments were conducted in triplicate, and the results are presented as mean values ± standard deviation (SD). Statistical comparisons between two groups were performed using the Student’s t-test. The threshold for statistical significance was set at *p < 0.05, **p < 0.01, and ***p < 0.001, with *p < 0.05 considered marginally significant, **p < 0.01 deemed highly significant, and ***p < 0.001 regarded as extremely significant. Conversely, results with *p > 0.05 were considered statistically non-significant (N.S.).

## 3 Results

### 3.1 Preparation and characterization of P(H)ZPAg NPs

**FIGURE 1:**
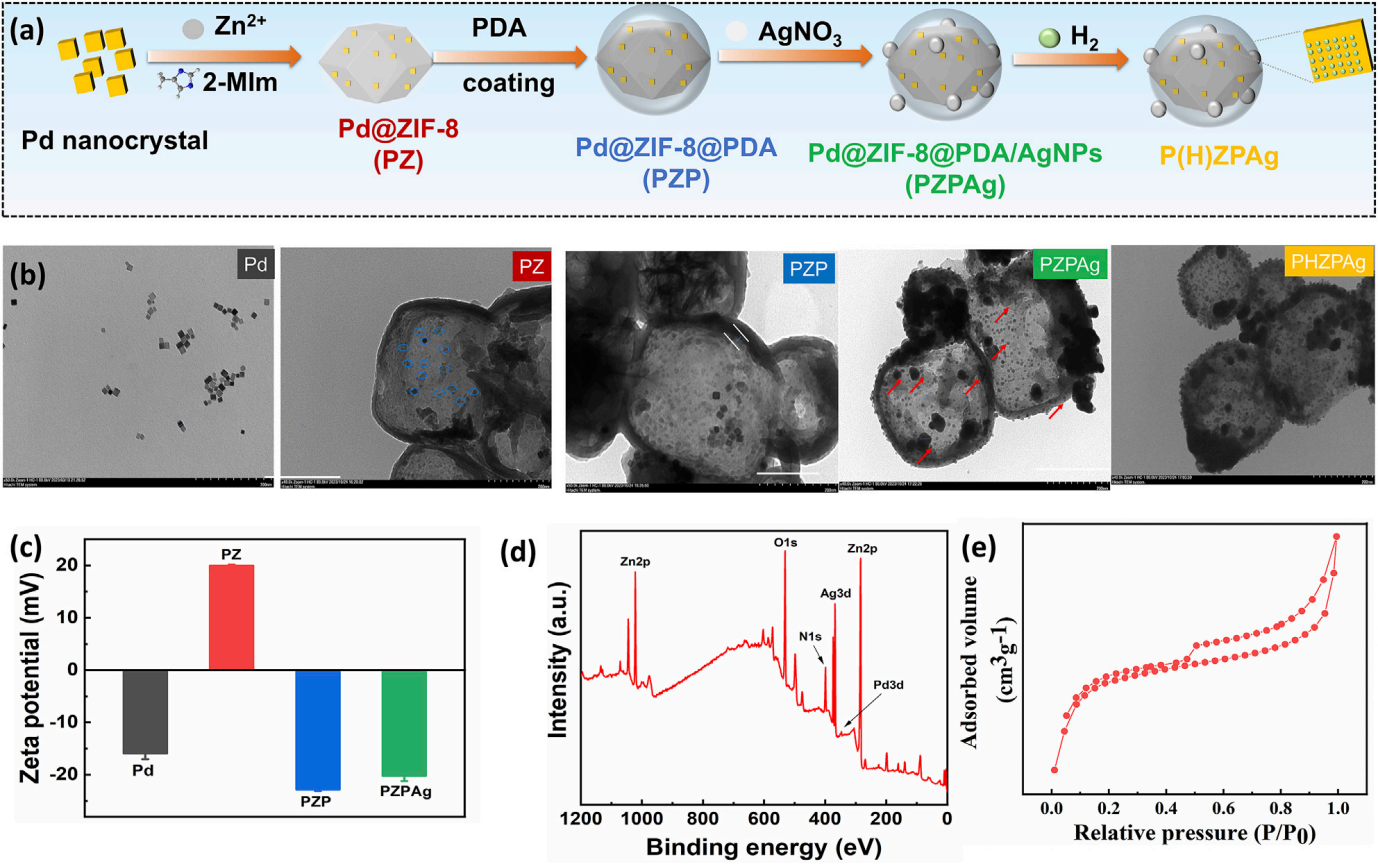
Preparation and characterization of PZPAg NPs. **(a)** Schematic illustration depicting the synthesis of P(H)ZPAg NPs. **(b)** TEM images and **(c)** zeta potential of Pd NPs, PZ NPs, PZP NPs and PZPAg NPs. **(d)** XPS spectra and **(e)** N_2_ adsorption–desorption isotherms of PZPAg NPs.

Palladium (Pd) is widely recognized for its high hydrogen storage capacity and excellent catalytic activity, particularly at the nanoscale (Li et al., 2014). Recent research has highlighted the potential of ZIF-8, a subclass of MOFs, in antibacterial applications due to their ability to combat bacterial infection, favorable biocompatibility, and acid-degradable properties (Yan et al., 2022). The synthesis of the P(H)ZPAg nanoplatform is outlined in Figure 1a. Initially, Pd nanoparticles (Pd NPs) were encapsulated within ZIF-8 through a one-pot reaction, creating a Pd@ZIF-8 (PZ) nanoparticles, which efficiently stores hydrogen. To enhance the functionality, a PDA shell was polymerized around the PZ structure (called Pd@ZIF-8/PDA, PZP NPs) through an *in-situ* polymerization of dopamine (Yang et al., 2021), providing stability and biocompatibility. Ag NPs were then integrated into the PDA layer via a redox reaction (Tong et al., 2020), forming Pd @ ZIF-8@PDA/Ag (PZPAg NPs). This combination endows the nanoplatform with additional antibacterial properties, as Ag^+^ ions are known to disrupt bacterial membranes. The finally P(H)ZPAg was synthesized by introducing hydrogen into PZPAg NPs solution using hydrogen-generation device. With the design, hydrogen-releasing from Pd NPs for *in situ* hydrogen therapy, the PDA shell exhibited photothermal bactericidal ability, and Ag^+^ and Zn^2+^ respectively released from AgNPs and ZIF-8 exhibited chemical antibacterial ability.

Transmission electron microscopy (TEM) images (Figure 1b) confirmed the cubic shape and smooth surface of Pd NPs, with an average diameter of approximately 10 nm. When incorporated into ZIF-8, the diameter of PZ NPs increased in size to 240 ± 16 nm. The successful loading of Pd NPs (blue circles) into ZIF-8 was further validated by TEM. Next, a PDA shell, approximately 10 nm thick, was polymerized around PZ NPs, forming a yolk-shell structure, as seen in Figure 1b. AgNPs were then added to the surface of PZP NPs via *in situ* reduction of Ag^+^ by PDA’s catechol groups, resulting in the formation of PZPAg nanohydride. TEM images confirmed the uniform distribution of AgNPs with an average size of 26 ± 3 nm. There was no difference in morphology for P(H)ZPAg after hydrogen loading.

Dynamic light scattering (DLS) measurements showed an increase in particle size following the PDA coating (Supplementary Figure S2), which was accompanied by a shift in zeta potential from +19.9 mV to −22.8 mV (Figure 1c). This suggests that the PDA layer successfully coated the PZ NPs. Additional characterization through X-ray photoelectron spectroscopy (XPS) showed a uniform distribution of elements including zinc (Zn), silver (Ag), palladium (Pd), nitrogen (N), and oxygen (O) throughout the PZPAg NPs (Figure 1d), confirming the successful synthesis of the hydride nanoparticles.

The porosity of the core-shell catalyst was further assessed by nitrogen sorption-desorption isotherms at 77 K. The isotherm exhibited a type IV pattern, with a sharp increase in nitrogen uptake at low relative pressures, indicating microporosity (Figure 1e). The Brunauer–Emmett–Teller (BET) surface area and pore volume were calculated to be 588.4 m^2^/g and 0.0.337 cm^3^/g, respectively (Supplementary Tables S1). The decrease in both surface area and pore volume can be attributed to PDA and AgNPs on the surface, which contributes significantly to the overall mass of the core-shell structure (Kasemset et al., 2017). The promising porous structural characteristics is beneficial for hydrogen loading.

### 3.2 Photothermal conversion efficiency of PZPAg NPs

**FIGURE 2:**
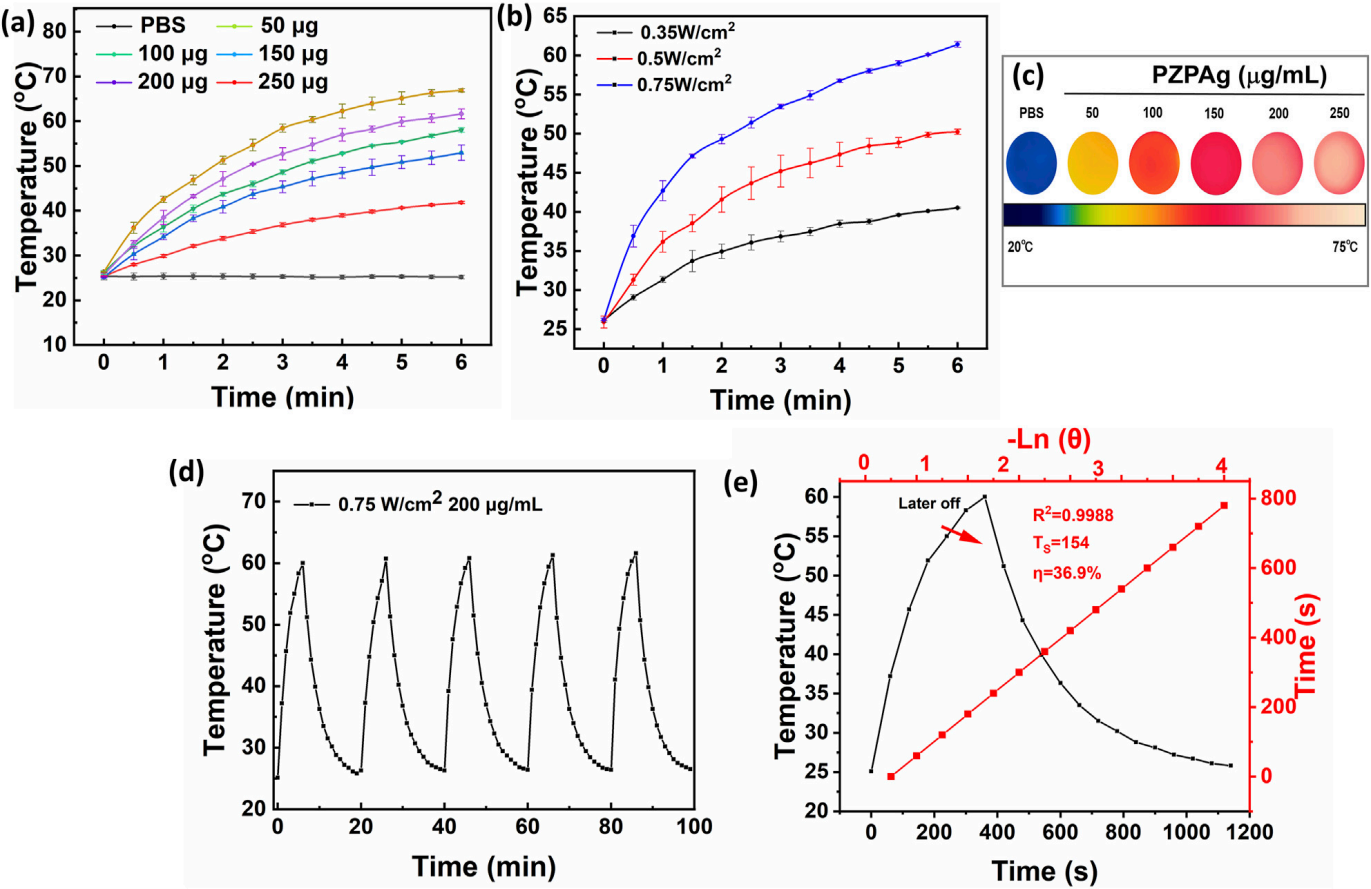
The photothermal properties of PZPAg NPs. **(a)** Temperature changes of PZPAg NPs solution with different concentrations (0, 50, 100, 150, 200, and 250 μg mL^−1^) under 808 nm laser irradiation (0.75 W cm^−2^) for 6 min. **(b)** Temperature curves of PZPAg NPs solution (200 μg mL^−1^) under 808 nm laser irradiation with various laser powers (0.35, 0.50 and 0.75 W cm^−2^). **(c)** Thermal images of PZPAg NPs at different concentrations after 6 min of 0.75 W cm^−2^ NIR irradiation. **(d)** Photothermal stability of PZPAg NPs at a concentration of 200 μg mL^−1^ over five irradiation cycles (808 nm, 0.75 W cm^−2^). **(e)** Heating−cooling curve and Plot of cooling time versus negative natural logarithm of the temperature driving force.

PDA is known for its broad light absorption spectrum, which allows it to efficiently convert absorbed energy into heat, making it an ideal candidate for photothermal therapy (Yang et al., 2021). The photothermal conversion efficiency of the PZPAg NPs was evaluated under 808 nm NIR laser irradiation. Initially, the temperature response of the PZPAg NPs solution was measured as a function of nanoparticle concentration, with a fixed power density of 0.75 W cm^−2^. As shown in Figure 2a, the temperature of the PZPAg NPs solution increased significantly upon exposure to 808 nm NIR light. For instance, a 250 μg/mL solution of PZPAg NPs heated from 26.1°C to 61.4°C within 6 min, demonstrating both the strong NIR light absorption and the concentration-dependent photothermal performance of the nanocomposite. The temperature increase was also tested at a fixed concentration of 200 μg/mL under varying NIR laser power densities. As shown in Figure 2b, the photothermal effect of PZPAg NPs intensified with higher laser power. At a power density of 0.75 W cm^−2^, the solution temperature increased by approximately 35.2°C in just 6 min, indicating the potential of PZPAg to disrupt bacterial membranes. Further real-time infrared thermal imaging (Figure 2c) confirmed that the heat generated by PZPAg NPs was sufficient to effectively kill bacteria.

Additionally, the photostability of PZPAg NPs was assessed by alternating the NIR laser irradiation cycles. As seen in Figure 2d, the temperature of the PZPAg solution increased by around 34.9°C after four irradiation cycles, demonstrating good reversibility and durability for repeated photothermal therapy applications. The photothermal conversion efficiency (η) of PZPAg NPs was calculated (Equation 1) to be 36.9%, based on the heating-cooling curve and the time constant obtained from the cooling period (Figure 2e). These results confirm the effective photothermal properties and stability of PZPAg NPs for potential therapeutic applications.

### 3.3 Releasing reductive hydrogen and scavenging reactive oxygen species (ROS) capacity of PHZAP NPs

Pd is well-known for its high hydrogen storage capacity, as hydrogen atoms can easily integrate into its crystal lattice, forming PdH nanohydride (Li et al., 2014). This property, coupled with its catalytic activity, makes Pd a valuable material for hydrogenation reactions, especially at the nanoscale. To evaluate the hydrogen release profile of P(H)ZPAg nanohydride, we utilized a methylene blue (MB) probe, a common method for detecting hydrogen release detection and quantification (Li et al., 2023). Typically, MB is combined with platinum (Pt) nanoparticles to enhance its hydrogenation capacity, allowing for rapid detection. The fading of the blue color in the MB solution indicates the reduction of MB, and the amount of hydrogen released can be quantified by measuring the decrease in absorbance at the characteristic MB peak (664 nm).

**FIGURE 3:**
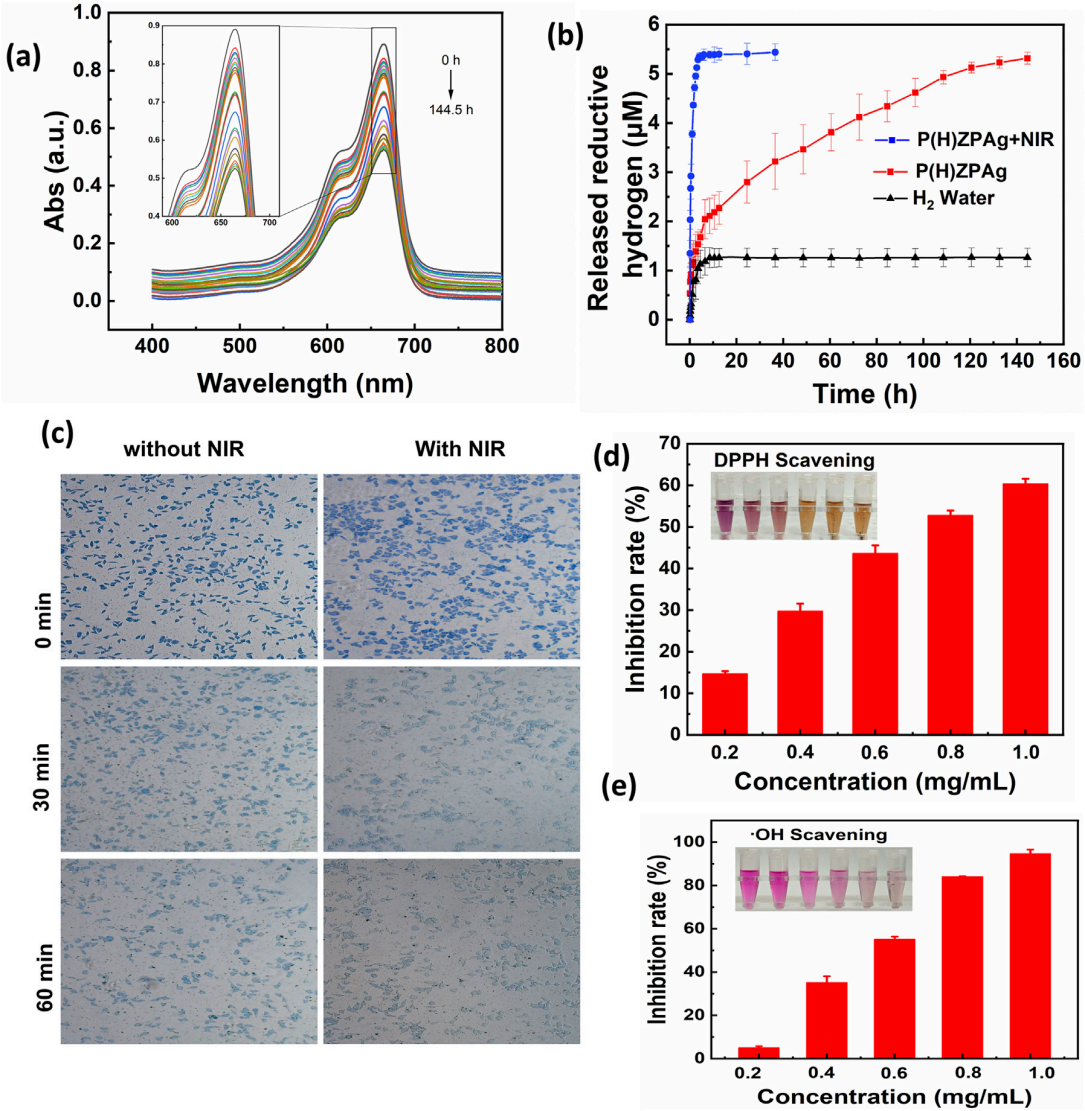
H_2_ release and antioxidant profile of P(H)ZPAg NPs. **(a)** Changes in UV-Vis spectra of MB incubated with P(H)ZPAg NPs at room temperature. **(b)** H_2_ release profile of H_2_ water, P(H)ZPAg NPs and P(H)ZPAg NPs with NIR irradiation. **(c)** Hydrogen production experiments of P(H)ZPAg NPs in L929 cells. **(d)** DPPH and **(e)** hydroxyl radicals scavenging activity of P(H)ZPAg NPs.

As shown in Figure 3a, the absorbance of MB at 664 nm gradually decreased when incubated with P(H)ZPAg nanohydride at 25°C, indicating a continuous release of hydrogen over time. The degree of color fading corresponds to the amount of hydrogen released, which was tracked using UV-Vis absorbance measurements. According to the standard curve (Supplementary Figure S1), the hydrogen release profile of P(H)ZPAg nanohydride was established (Figure 3b). The results demonstrated that hydrogen was released rapidly during the first 5 h, followed by a slower release over the next 19 h, totaling about 5 μM of hydrogen at 25°C. Interestingly, the reductive capacity of hydrogen within P(H)ZPAg was significantly higher than that of hydrogen gas itself (Figure 3b). Furthermore, the hydrogen release rate from P(H)ZPAg nanohydride could be enhanced by applying NIR laser irradiation (Figure 3b). This suggests that on-demand hydrogen release can be precisely controlled by adjusting the NIR laser power density and irradiation time, which is crucial for optimizing hydrogen’s therapeutic effects. These findings highlight the potential of P(H)ZPAg nanohydride as a versatile and controllable hydrogen delivery platform for therapeutic applications.

The intracellular hydrogen release from P(H)ZPAg nanohydride was further investigated using methylene blue-platinum (MB-Pt) as a probe (Chen et al., 2021). As illustrated in Figure 3c, following treatment with P(H)ZPAg nanohydride, the blue coloration of the cells progressively diminished over time. This fading indicates a significant increase in hydrogen (H_2_) release within the cells, attributed to the reduction of MB by the released H_2_ under the catalytic action of platinum (Pt). These observations provide clear evidence of the time-dependent hydrogen generation capability of P(H)ZPAg nanohydride in a cellular environment.

Excessive oxidative stress contributes significantly to tissue damage during bacterial infections, often leading to necrosis and impaired wound healing (Wang G. et al., 2023). Both molecular hydrogen (Jiang et al., 2024) and PDA (Yang et al., 2021) are recognized for their antioxidant properties. To evaluate the antioxidative potential of P(H)ZPAg NPs, we conducted tests using several representative ROS, including DPPH, and hydroxyl radicals (•OH). As shown in Figure 3d, the purple color of the DPPH solution gradually faded with increasing concentrations of PHZAP NPs, and the DPPH scavenging rate (Equation 2) reached 60.4% at 1 mg/mL. This indicates a significant ROS-neutralizing effect. The •OH-scavenging activity of P(H)ZPAg NPs was also examined, revealing a substantial reduction in •OH levels. At a concentration of 1 mg/mL, the •OH-scavenging rate (Equation 3) reached 94.5%, as evidenced by the visible fading of the solution (Figure 3e). Collectively, these results highlight the broad-spectrum ROS-scavenging capacity of P(H)ZPAg NPs, emphasizing their potential in mitigating oxidative stress-related tissue damage during infection and promoting wound healing.

### 3.4 Antimicrobial properties of P(H)ZPAg NPs

**FIGURE 4:**
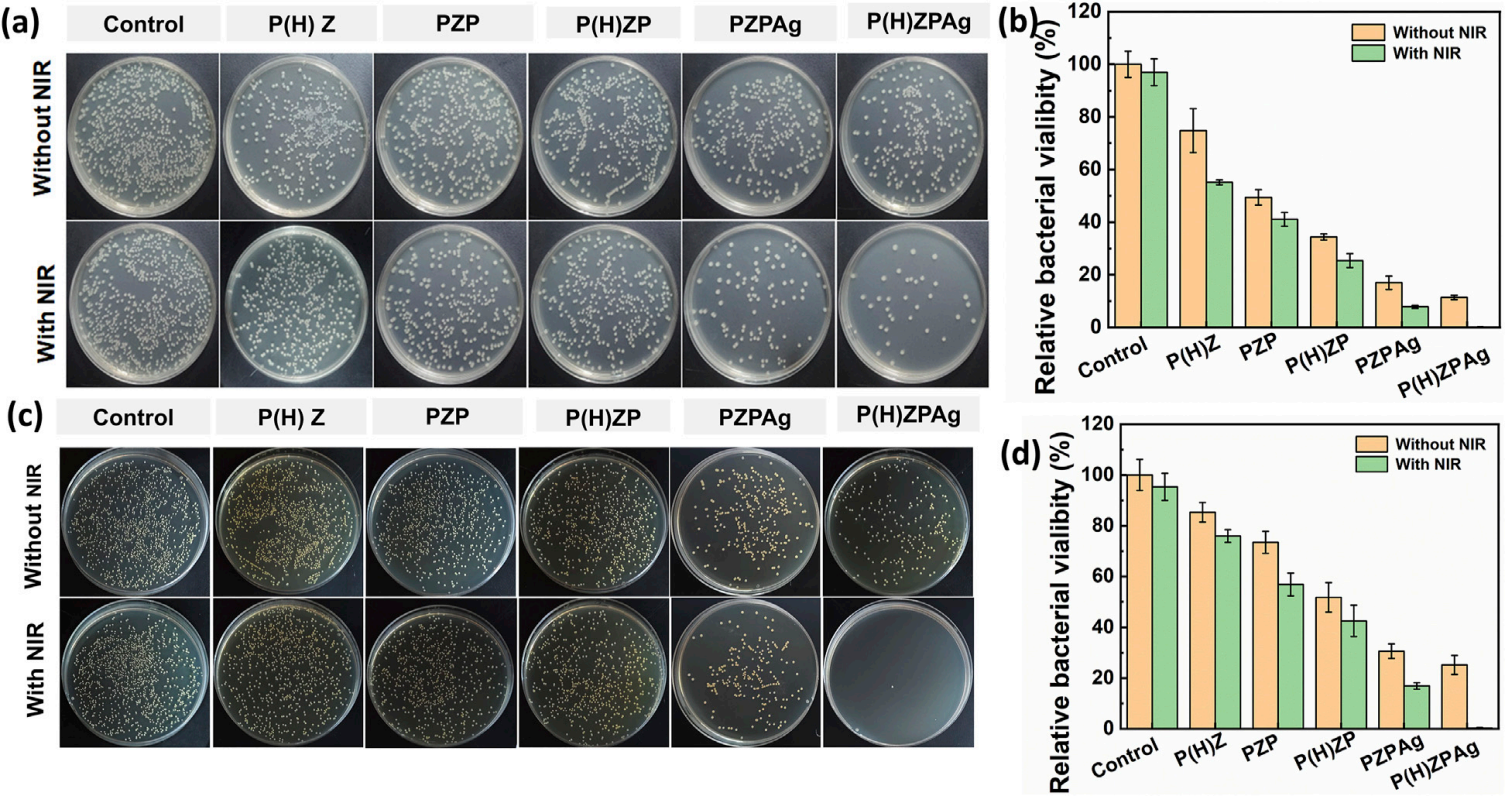
Evaluation of the antibacterial activity of P(H)ZPAg NPs. Plate counting assays of *S. aureus* **(a)** and f *E. coli* **(c)** incubation with different treatment. Relative percentages of bacterial remaining on *S. aureus* **(b)** and *E. coli* **(d)** after treatments.

Chronic wounds are often complicated by bacterial infections, which exacerbate inflammation, promote pus formation, and delay the healing process (Falcone et al., 2021). Given the hydrogen-releasing properties of P(H)ZPAg nanohydride and its ability to convert light into heat, we investigated its antibacterial performance *in vitro*, targeting both Gram-positive *S. aureus* and Gram-negative *E. coli* using a plate counting method. As shown in Figures 4a,b, when tested against *S. aureus*, P(H)Z alone exhibited limited antibacterial activity, with a bacterial survival rate of 85.3%, likely due to the mild effect of the hydrogen released. However, when PDA was added to the P(H)Z structure, the antibacterial activity improved, particularly when exposed to NIR light. This enhancement suggests a synergistic effect between hydrogen release and photothermal PTT. The addition of AgNPs further decreased bacterial survival, likely due to the combined effects of hydrogen release and the antibacterial action of Ag^+^ ions. Notably, after 808 nm NIR irradiation, the bacteriostatic effect of P(H)ZPAg reached 99.3% In contrast, P(H)ZPAg without hydrogen release showed a less significant reduction in bacterial survival, indicating that hydrogen release enhanced the antibacterial efficacy by increasing bacterial membrane permeability. A similar trend was observed with *E. coli*, where P(H)ZPAg + NIR achieved a sterilization rate of 99.8% (Figures 4c,d). The combined effects of hydrogen and heat from PTT likely disrupted bacterial membranes, facilitating the entry of Ag^+^ ions, which contributed to bacterial death.

**FIGURE 5:**
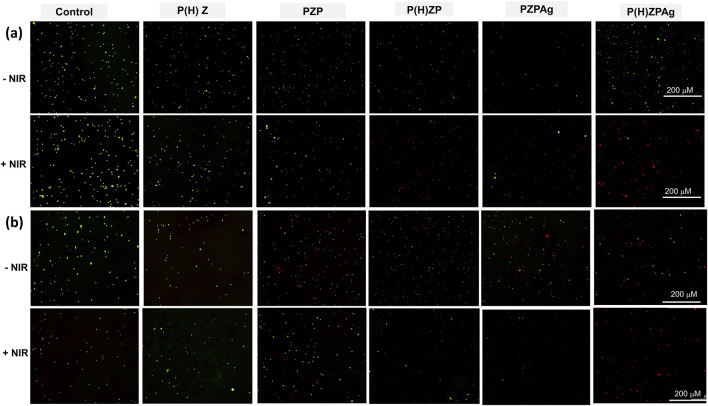
Live/dead co-staining of *S. aureus* **(a)** and *E. coli* **(b)** after treatments. Scale bars: 200 µm.

Further validation through live-dead staining using DMAO/EthD-III demonstrated the effectiveness of P(H)ZPAg + NIR treatment (Figure 5). Bacteria in untreated groups predominantly displayed green fluorescence, indicating live cells, while those treated with P(H)ZPAg + NIR showed mainly red fluorescence, indicative of dead bacteria. These findings confirm that the multimodal approach—combining PTT, hydrogen release, and Ag^+^ ions—enhances the antibacterial effect, offering a promising strategy for wound healing.

### 3.5 *In Vivo* wound healing analysis of P(H)ZPAg NPs

**FIGURE 6:**
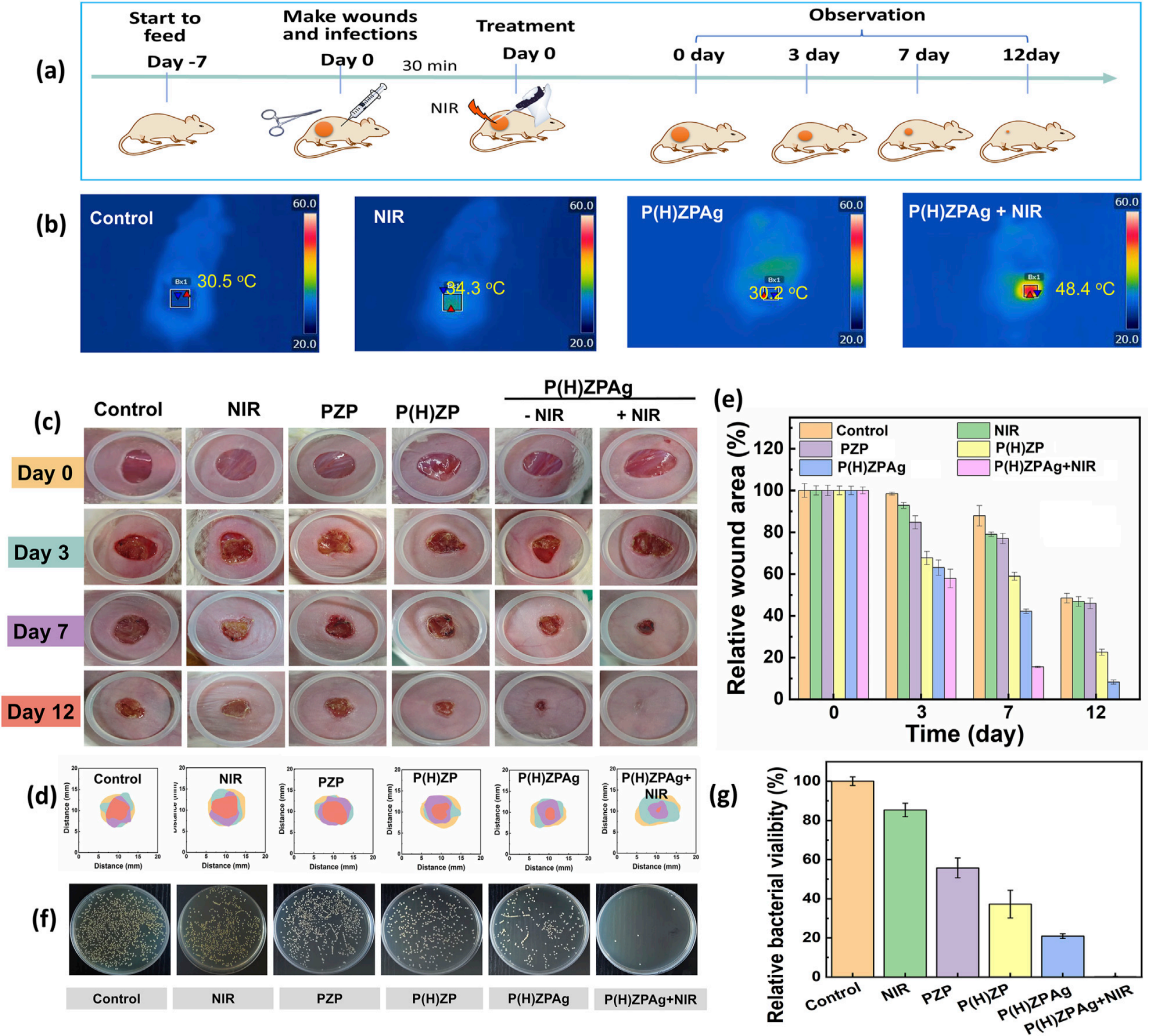
*In vivo* therapeutic efficacy of P(H)ZPAg was evaluated using a *S. aureus-*infected rat wound model. **(a)** Schematic illustration for the establishment of *in vivo* bacterial infections model and subsequent treatment regime. **(b)** Infrared thermograms and recordings of mice under laser irradiation (0.75 W cm^−2^) for 6 min. **(c)** Digital images and **(d)** physical analog maps, and **(e)** area quantification of area on different days underwent different treatment. **(f)** Photographs of bacterial colonies and **(g)** colony quantification after various treatments.

Building upon the promising *in vitro* antibacterial results, the *in vivo* therapeutic efficacy of P(H)ZPAg was evaluated using a *S. aureus* -infected rat wound model. The experimental setup for the *in vivo* studies is depicted in Figure 6a. A 5 mm diameter round wound was created on the back of mice, followed by inoculation of *S. aureus* (1 × 10^7^ CFU) into the fresh wounds. After 2 days of incubation, an infected wound model was established. The animals were then treated with different formulations, including PBS, NIR, PZP, P(H)ZP, P(H)ZPAg, and P(H)ZPAg combined with NIR irradiation, to evaluate their antibacterial effects on the open wounds. First, the *in vivo* photothermal effect of P(H)ZPAg was assessed by monitoring the temperature at the wound sites during laser treatment. As shown in Figure 6B, the wound temperature increased sharply from 30.5°C to 48.4°C within 6 min of 808 nm laser irradiation (0.75 W cm^−2^), whereas the PBS-treated wound temperature only reached 34.3°C. This result supports the hypothesis that P(H)ZPAg, under NIR irradiation, function as a potent photothermal antibacterial agent *in vivo*. Importantly, no significant damage to the surrounding skin tissue was observed during the treatment.

Macroscopic examination of wound healing was performed by capturing images at various time points and measuring the wound closure rates (Figure 6c). On day 3, scars were visible in all treatment groups, except for the PBS and NIR group. Over time, the scars darkened and reduced in size, indicating that bacterial infection was effectively controlled and wound healing was underway. The wound closure in the P(H)ZPAg and P(H)ZPAg groups showed significantly better healing compared to the PBS, PZP and P(H)ZP groups. By day 12, the P(H)ZPAg + NIR group showed complete wound closure, with no visible scar, indicating that the synergistic treatment led to rapid and effective sterilization. Quantitative analysis of wound area was also conducted (Figures 6d.e). After 7 days of treatment, the relative wound areas in the PBS, NIR, PZP, P(H)ZP, P(H)ZPAg, and P(H)ZPAg + NIR groups decreased to 87.8%, 80.1%, 77.1%, 58.9%, 42.1%, and 15.5%, respectively. Remarkably, by day 12, the wound area in the P(H)ZPAg + NIR group reduced to just 0.4% of the initial size, demonstrating the outstanding therapeutic potential of P(H)ZPAg NPs in promoting wound healing through the combined effects of PTT, hydrogen release, and AgNPs under NIR irradiation.

In addition, to assess the antibacterial efficacy *in vivo*, wound tissues were harvested on day 5 and the bacterial load was quantified using the plate counting method. As shown in Figures 6F,G, the lowest bacterial survival (0.1%) was found in the PHZAP + NIR treatment group, significantly lower than in the positive control and PHZAP-only groups. These results suggest that PHZAP NPs, when coupled with NIR irradiation, effectively eradicated *S. aureu*s infection *in vivo* through synergistic antibacterial effects, leading to enhanced wound healing. Thus, PHZAP NPs represent a promising candidate for the treatment of infected chronic wounds.

### 3.6 *In vivo* histopathological analysis

**FIGURE 7:**
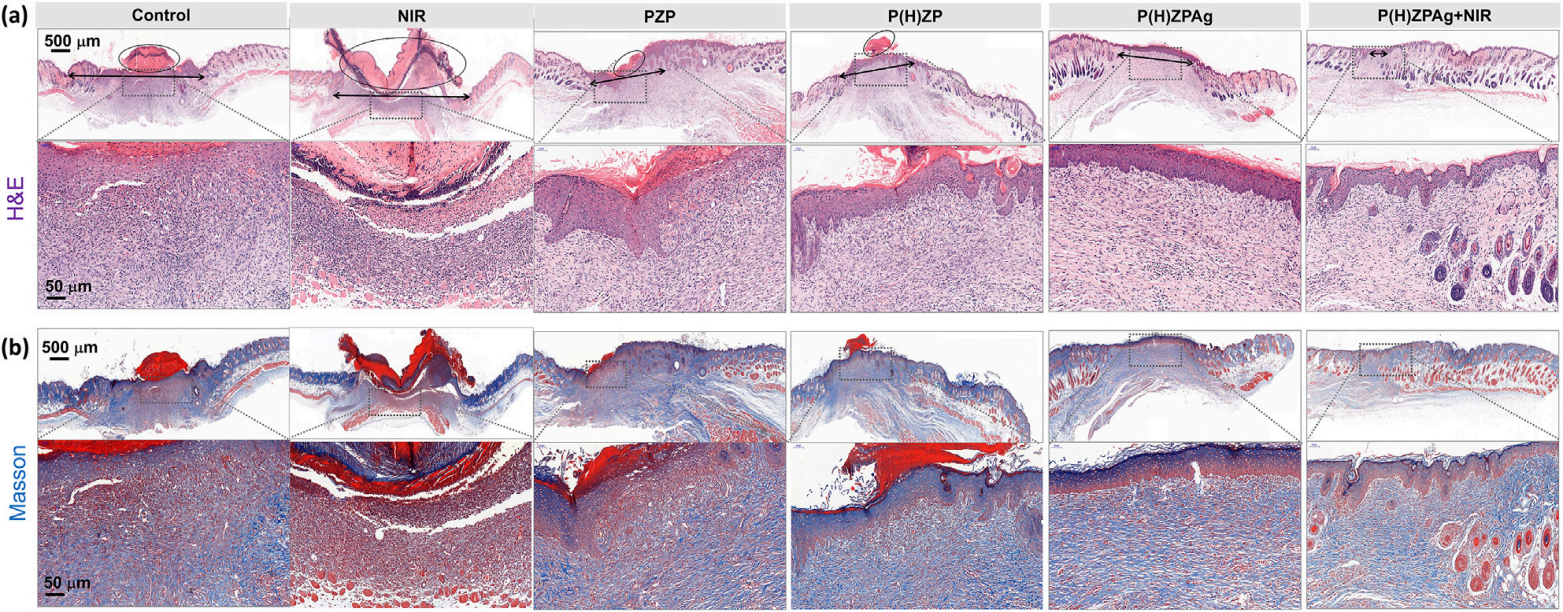
*In Vivo* Histopathological Analysis after various treatments. **(a)** H&E staining and **(b)** Masson’s trichrome-stained images of various treated abscess tissues. Scale bars: 500 and 50 µm.

To assess structural changes in the epidermis and collagen deposition during wound healing, histopathological analyses were conducted on wound tissues harvested on day 12 using hematoxylin and eosin (H&E) and Masson’s trichrome staining. As depicted in Figure 7a, the control group (I), NIR group (II), the PZP-treated group (III), and the P(H)ZP(IV) displayed substantial areas of unhealed tissue covered by scabs. Notably, large epithelial gaps (marked by black circles) and necrotic foci were observed, indicative of delayed healing and persistent tissue damage. In stark contrast, the P(H)ZPAg + NIR-treated group (VI) exhibited nearly complete epithelial regeneration, characterized by well-formed granulation tissue and the presence of skin appendages, underscoring its exceptional regenerative capacity.

Collagen deposition, a critical factor in wound healing, plays a pivotal role in maintaining structural integrity, facilitating cellular migration, and supporting the formation of a new extracellular matrix. As illustrated in Figure 7b, the P(H)ZPAg + NIR group (VI) demonstrated a more organized and dense collagen arrangement compared to other groups. This enhanced collagen organization is essential for effective extracellular matrix remodeling, thereby promoting robust skin tissue regeneration. Collectively, these findings highlight the superior wound-healing efficacy of the P(H)ZPAg + NIR treatment, further validating the remarkable potential of this multimodal synergistic therapeutic approach.

### 3.7 Evaluation of biocompatibility of P(H)ZPAg NPs

**FIGURE 8:**
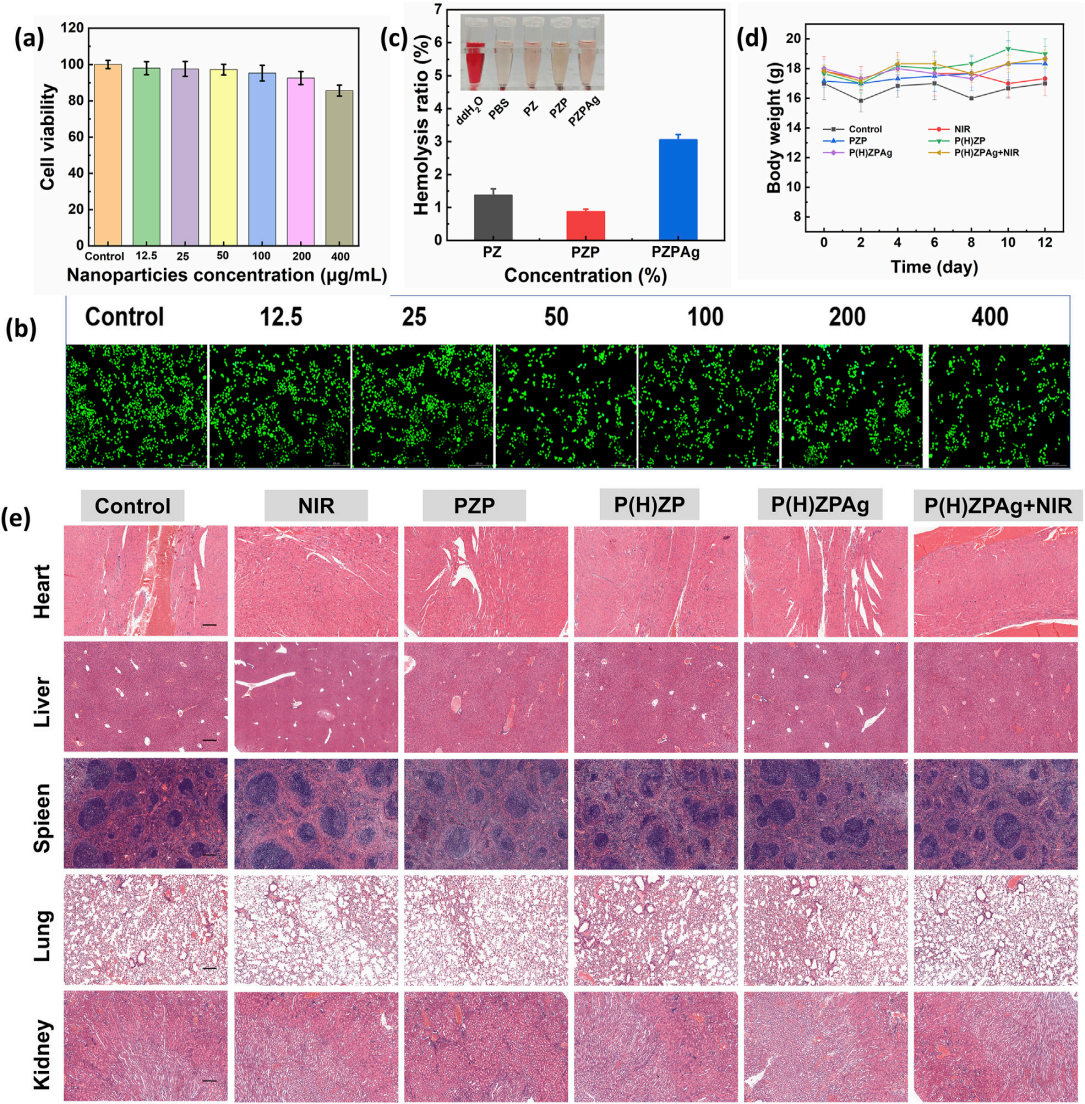
Evaluation of biocompatibility of P(H)ZPAg NPs. **(a)** MTT assays and **(b)** LIVE/DEAD viability staining of P(H)ZPAg NPs with different concentrations (μg mL^−1^). Scale bars: 200 µm. **(c)** Relative Hemolysis ratios of water, PBS, and Noise **(d)** Body weight change curves of mice in different groups as a function of time. **(e)** H&E-stained tissue slices from major organs (heart, liver, spleen, lung and kidney) of different treatment groups. Scale bars: 200 µm.

Biocompatibility is crucial for the successful *in vivo* application of therapeutic materials (Chandy, 2020). To assess the cellular compatibility of P(H)ZPAg, a series of tests were conducted using fibroblast cells (L929), including MTT assays and LIVE/DEAD viability staining. As shown in Figure 8a, cell viability remained above 85% across a concentration range from 12.5 to 400 μg/mL, indicating that P(H)ZPAg NPs did not exhibit significant cytotoxicity. LIVE/DEAD staining further confirmed this, with green fluorescence marking viable cells and red fluorescence indicating dead cells (Figure 8b). Very few red-stained cells were observed, demonstrating the excellent biocompatibility of P(H)ZPAg NPs.

Additionally, hemolysis assays were performed to evaluate potential toxicity to red blood cells. Unlike the positive control group, which showed complete erythrocyte lysis, the erythrocyte suspension in the P(H)ZPAg NPs group remained clear with only minimal hemolysis (3.0%), well below the internationally accepted threshold of 5% for hemolytic toxicity (Figure 8c). These results underscore the low hemolytic potential of PHZAP NPs.

*In vivo*, the safety of PHZAP NPs was further confirmed through body weight monitoring (Figure 8d) and histological analysis of major organs, including the heart, liver, spleen, lungs, and kidneys, after treatment. Hematoxylin and eosin (H&E) staining (Figure 8e) revealed no adverse effects, and blood biochemistry tests indicated that all key blood markers remained within normal ranges (Supplementary Figure S3). Together, these findings validate the excellent biocompatibility and safety profile of P(H)ZPAg NPs, highlighting their potential for antibacterial and wound-healing applications in clinical settings.

## 4 Discussion

In this study, a multifunctional antibacterial therapy nanoplatform based on PdH-hydride MOFs was developed in the treatment of bacterial infections associated with skin damage. The multifunctional P(H)ZPAg represents a significant integration of hydrogen release, PTT, and silver AgNP therapy, addressing several limitations of conventional treatments. By leveraging the synergistic effects of these three therapeutic modalities, we observed a marked enhancement in antibacterial efficacy, as evidenced by the substantial reduction in bacterial viability both *in vitro* and *in vivo*.

Hydrogen, known for its strong reducing properties and biocompatibility, has been shown to disrupt bacterial membrane permeability, facilitating the entry of antibacterial agents and promoting cellular content leakage (Wang T.-Y. et al., 2023; Wang Z. et al., 2023; Benoit Stéphane et al., 2020; Li et al., 2023). However, the low tissue permeability and poor solubility of hydrogen have historically limited its therapeutic utility (Jiang et al., 2024). By encapsulating PdH within ZIF-8 and utilizing NIR irradiation, we achieved controlled hydrogen release, which significantly enhanced the bactericidal effects of the nanoplatform. PTT is another critical component of the P(H)ZPAg nanoplatform. The use of PDA nanoparticles, which exhibit excellent photothermal conversion properties, allowed for effective heat generation upon NIR irradiation (Yang et al., 2021). This hyperthermia-induced disruption of bacterial cell membranes, protein denaturation, and DNA damage resulted in significant bacterial cell death (Chen et al., 2020). The combination of hydrogen release and PTT further amplified the bactericidal effects, as the elevated temperatures facilitated the diffusion of hydrogen into bacterial cells, thereby enhancing the overall antibacterial performance. AgNPs were also integrated into the P(H)ZPAg nanoplatform to leverage their broad-spectrum antimicrobial activity. AgNPs are known to release metal ions that interact with bacterial proteins and enzymes, generating ROS that cause structural damage to bacterial cell walls and membranes (Tong et al., 2020). The deposition of AgNPs onto the PDA surface through coordination forces and *in situ* reduction by catechol groups of PDA ensured a stable and controlled release of Ag+ ions, further contributing to the enhanced antibacterial efficacy.

The *in vitro* results demonstrated that P(H)ZPAg NPs exhibited antibacterial activity against both Gram-positive and Gram-negative bacteria, including *E. coli* and *S. aureus*. The synergistic effect of hydrogen release, PTT, and Ag + ions led to a significant reduction in bacterial viability. The *in vivo* results further corroborated the efficacy of P(H)ZPAg NPs. In the S. aureus-infected rat model, the PHZAP + NIR treatment group showed rapid and effective wound healing, with complete wound closure observed within 12 days. Histological evaluations confirmed minimal bacterial presence and no signs of tissue damage, underscoring the safety and biocompatibility of the nanoplatform. These results are demonstrating the therapeutic potential of combined therapies in wound healing. These findings highlight the potential of P(H)ZPAg NPs as a promising strategy for treating chronic, infection-induced wounds, offering a highly effective and safe platform for future antibacterial therapies. Looking ahead, several future research directions are essential to advance the clinical translation of the P(H)ZPAg nanoplatform. Comparative studies against current clinical standards, such as antibiotic therapies and commercial silver dressings, are necessary to benchmark its therapeutic advantages. Comprehensive biosafety evaluations, including long-term toxicity and immunogenicity assessments, will be conducted to ensure clinical safety. Mechanistic investigations using molecular profiling techniques will elucidate the pathways underlying the observed synergistic antibacterial effects. Overall, the findings suggest that PHZAP NPs represent a promising strategy for treating chronic, infection-induced wounds, offering a highly effective and safe platform for future antibacterial therapies.

## Data Availability

The original contributions presented in the study are included in the article/Supplementary Material, further inquiries can be directed to the corresponding author.
