# Supplementary material for: Multifunctional PdH-hydride MOFs for synergistic hydrogen and photothermal antibacterial therapy in accelerated wound healing
Source: Front Pharmacol. 2025 Apr 30;16:1587890. doi: 10.3389/fphar.2025.1587890 (PMC12075260; doi:10.3389/fphar.2025.1587890)
Supplement: Supplementary file 1 [file DataSheet1.docx]

Supplementary Material

# Supplementary Figures and Tables

## Supplementary Tables

**Supplementary Tables 1.** Surface areas and pore volumes calculated by the BET equation

| **Samples** | **BET surface area**  **(m^2^/g)** | **Pore volume**  **(m^2^/g)** | **pore diameter**  **(nm)** |
| --- | --- | --- | --- |
| PZ | 1620.1±50.2 | 0.652±0.06 | 2.62±0.21 |
| PZPAg | 588.3±32.5 | 0.337±0.04 | 2.29±0.32 |

## Supplementary Figures

##
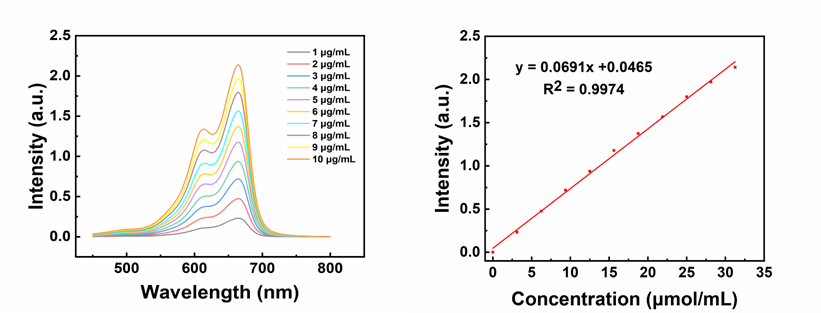


**Supplementary Figure 1.** Changes in UV-Vis spectra of aqueous solution with different concentrations MB and the plotting the standard curve of MB.





**Supplementary Figure 2.** The size distribution f Pd NPs, PZ NPs, PZP NPs and PZPAg NPs.


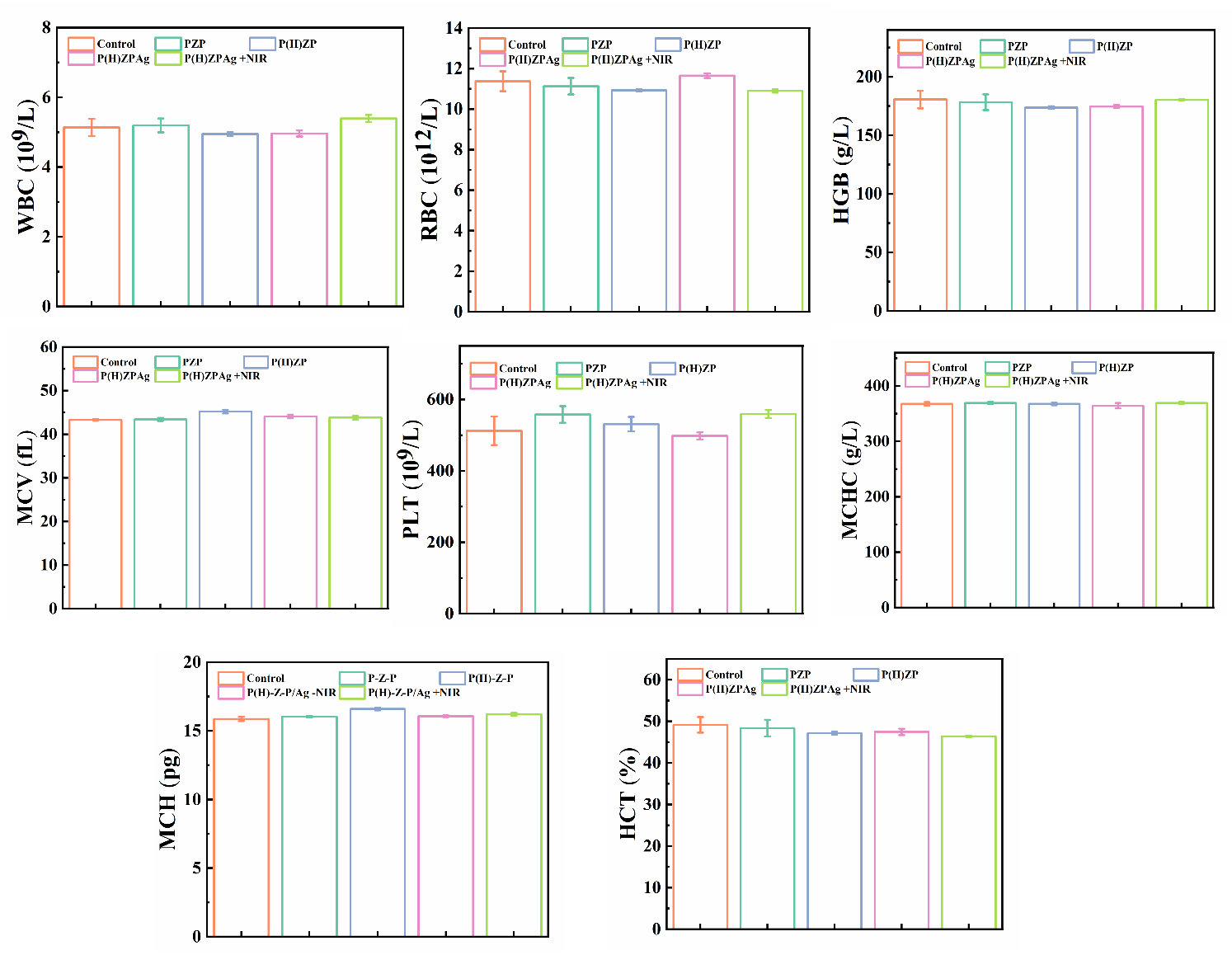


**Supplementary Figure 3.** Blood biochemistry tests of different treatment groups during *in vivo* wound healing experiment.
